# Low rates of myocardial fibrosis and ventricular arrhythmias in recreational athletes after SARS-CoV-2 infection

**DOI:** 10.3389/fcvm.2024.1372028

**Published:** 2024-04-02

**Authors:** Hielko Miljoen, Kasper Favere, Caroline Van De Heyning, Ben Corteville, Christophe Dausin, Lieven Herbots, Tom Teulingkx, Youri Bekhuis, Malou Lyssens, Jan Bogaert, Hein Heidbuchel, Guido Claessen

**Affiliations:** ^1^ Department of Cardiology, Antwerp University Hospital, Antwerp, Belgium; ^2^ Research Group Cardiovascular Diseases, Department GENCOR, University of Antwerp, Antwerp, Belgium; ^3^ Department of Internal Medicine, Ghent University, Ghent, Belgium; ^4^ Department of Cardiology, Jan Palfijn Hospital, Ghent, Belgium; ^5^ Exercise Physiology Research Group, Department of Movement Sciences, KU Leuven, Leuven, Belgium; ^6^ Department of Cardiology, Hartcentrum, Jessa Hospital, Hasselt, Belgium; ^7^ Faculty of Medicine and Life Sciences, Hasselt University, Hasselt, Belgium; ^8^ Artsenpraktijk Spikdorenveld, Westerlo, Belgium; ^9^ Department of Cardiology, Leuven University Hospitals, Leuven, Belgium; ^10^ Department of Cardiovascular Sciences, Faculty of Medicine, KU Leuven, Leuven, Belgium; ^11^ Department of Imaging and Pathology, KU Leuven, Leuven, Belgium; ^12^ Department of Radiology, University Hospitals Leuven, Leuven, Belgium

**Keywords:** COVID-19, athlete, myocarditis, arrhythmias, recreational

## Abstract

**Introduction:**

High rates of cardiac involvement were reported in the beginning of the coronavirus disease 2019 (COVID-19) pandemic. This led to anxiety in the athletic population. The current study was set up to assess the prevalence of myocardial fibrosis and ventricular arrhythmias in recreational athletes with the recent severe acute respiratory syndrome coronavirus 2 (SARS-CoV-2) infection.

**Methods:**

Consecutive adult recreational athletes (≥18 years old, ≥4 h of mixed type or endurance sports/week) underwent systematic cardiac evaluation after a prior confirmed COVID-19 infection. Evaluation included clinical history, electrocardiogram (ECG), 5-day Holter monitoring, and cardiac magnetic resonance (CMR) imaging with simultaneous measurement of high-sensitive cardiac Troponin I. Data from asymptomatic or mildly symptomatic athletes (Group 1) were compared with those with moderate to severe symptoms (Groups 2–3). Furthermore, a comparison with a historical control group of athletes without COVID-19 (Master@Heart) was made.

**Results:**

In total, 35 athletes (18 Group 1, 10 female, 36.9 ± 2.2 years, mean 143 ± 20 days following diagnosis) were evaluated. The baseline characteristics for the Group 1 and Groups 2–3 athletes were similar. None of the athletes showed overt myocarditis on CMR based on the updated Lake Louise criteria for diagnosis of myocarditis. The prevalence of non-ischemic late gadolinium enhancement [1 (6%) Group 1 vs. 2 (12%) Groups 2–3; *p* = 0.603] or ventricular arrhythmias [1 Group 1 athlete showed non-sustained ventricular tachycardia (vs. 0 in Groups 2–3: *p* = 1.000)] were not statistically different between the groups. When the male athletes were compared with the Master@Heart athletes, again no differences regarding these criteria were found.

**Conclusion:**

In our series of recreational athletes with prior confirmed COVID-19, we found no evidence of ongoing myocarditis, and no more detection of fibrosis or ventricular arrhythmias than in a comparable athletic pre-COVID cohort. This points to a much lower cardiac involvement of COVID-19 in athletes than originally suggested.

## Introduction

In early 2020, immediately following the first reports of severe acute respiratory syndrome coronavirus-2 (SARS-CoV-2)-induced coronavirus disease (COVID)-19, signs of acute cardiac injury or myocarditis were reported to be seen in between 7.2% and 40.9% of (hospitalized) patients (1). These findings were confirmed in non-hospitalized patients, with cardiovascular magnetic resonance (CMR) studies showing cardiac involvement in up to (and even over) 50% of recovered patients, including a deterioration of ejection fraction (2, 3).

Subsequently, a small series of 26 athletes (15 male, mean age 19.5 years) who suffered from COVID-19 (12 of them symptomatic) was reported (4). Four athletes had CMR signs of myocarditis (15%), and eight additional athletes demonstrated late gadolinium enhancement (LGE) on CMR (31%). These observations led to a high level of anxiety in the athletic population, for both myocarditis and the presence of LGE after myocarditis have been associated with sudden cardiac death in athletes and non-athletes (5, 6). The frequent occurrence of palpitations after SARS-CoV-2 infection raised further concern, for the fear of ventricular arrhythmias (7).

This and other series were hindered by the non-uniform and often single modality (only CMR) evaluation of the study subjects, while consensus documents underscore the importance of multimodality evaluation of athletes with suspected pathology (8, 9). Therefore, we set up the current study to systematically assess the prevalence of myocardial inflammation, fibrosis, and arrhythmias in athletes after SARS-CoV-2 infection.

## Methods

We used the “strengthening of the reporting of observational studies in the epidemiology cohort” (STROBE) checklist when writing this report (10). Approval of the local ethics committee was obtained. The trial was registered at www.clinicaltrials.gov (NCT04726150). All participants gave their written informed consent.

An “athlete” was defined as someone who performed endurance or mixed-type sports (11) for at least 4 h per week (12) and had done so for at least 5 consecutive years. For inclusion, SARS-CoV-2 infection had to be proven by polymerase chain reaction (PCR), serology, or chest computed tomography. The evaluation was performed at least 1 month after the onset of symptoms or the first positive polymerase chain reaction test.

Various sports confederations and their physicians were made aware of the project via email or word of mouth. Athletes who were willing to participate were directed toward a study website (www.covidex.be). This was set up in agreement with the most recent General Data Protection Regulation (GDPR) guidelines. Upon self-registration (through filling in name, date of birth, and email address) the secured online application on this website generated a unique code. This code could then be used by the athletes to gain access to a screening questionnaire via a separate link on the website. In this manner, the questionnaire data were coded and not directly linked to the personal data. The screening questionnaire was provided by REDCap, a web-based, electronic data capture tool for research studies (https://gbiomed.kuleuven.be/english/IT/it-solutions/Redcap) (13, 14). All research data were coded and stored in REDCap in accordance with GDPR.

Informed consent was obtained in two stages. The first stage consisted of a digital consent, stating that the athlete consented to be potentially contacted by the study office and with the use of his or her coded data for scientific purposes. Contact data for potential questions or withdrawal of consent was made available on the website. The second stage consisted of an in-person consent with the investigators. This stage applied only to those randomized to undergo further testing.

Exclusion criteria were known prior cardiac fibrosis, history of ventricular arrhythmias, known or newly diagnosed coronary artery disease, allergy or contraindications for gadolinium contrast, claustrophobia, and unwillingness or impossibility to give informed consent. Prior vaccination was not considered an exclusion criterion.

Three groups of patients were defined:

• Group 1: Asymptomatic or mildly symptomatic individuals. Mild symptoms included anosmia, ageusia, headache, mild fatigue, fever ≤3 days, myalgias ≤3 days, mild upper respiratory tract illness, and mild gastrointestinal illness.
• Group 2: Moderate symptoms and/or cardiac symptoms. Moderate symptoms included at least two of: persistent fever ≥4 days, chills ≥4 days, myalgias ≥4 days, lethargy impairing activities of daily living ≥4 days, dyspnea during activities of daily living ≥4 days, and chest tightness ≥4 days. Cardiac symptoms included dyspnea, exercise intolerance, chest tightness, dizziness, (pre)syncope, and (new onset) palpitations.
• Group 3: Any hospitalization for COVID-19 (whether or not on the intensive care unit).

In the first step, individuals not fulfilling the inclusion criteria and/or having exclusion criteria were excluded. Per protocol, all Group 3 subjects were included. Subsequently, self-registered athletes were randomly chosen for complete evaluation according to study protocol based on the number of available study slots in the central study site that needed to perform the CMR. Capacity was limited owing to pandemic-related restrictions and a high demand for CMR within a clinical context. The study evaluation comprised a thorough medical history, clinical examination, 12-lead electrocardiogram (ECG), laboratory testing for high-sensitive Troponin I (hsTnI; Atellica, Siemens®), a long-term (≥5 days) rhythm recorder (Rooti Rx, Healthstim), and a CMR.

Power calculations led us to aim for 73 inclusions, based on the 5% prevalence of non-ischemic fibrosis on CMR in our available athlete cohort (without COVID-19) (15) vs. a previously reported prevalence of at least 20% (as a conservative estimate) in COVID-19 athletes (3, 4). This sample size was calculated to achieve a power of 0.8 and a confidence level of 0.95.

The primary outcome measures were as follows:

• the proportion of athletes with positive updated Lake Louise (ULL) criteria (16) on CMR and
• the arrhythmic burden on Holter, defined as the average number of atrial and ventricular premature beats over 24 h.

Secondary endpoints consisted of persistent symptoms, ECG findings, and cardiac markers [high-sensitive cardiac Troponin I (hsTnI)].

As mentioned, findings of the present study could be compared with those of an available cohort of athletes included in the Master@Heart trial (17). That study included male endurance athletes between 45 and 70 years of age who performed either ≥6 h per week of running or ≥8 h per week of cycling with a control group of sedentary men (≤3 h of exercise per week). The subjects with cardiac risk factors were stringently excluded. The database consisted of 558 individuals at the time of analysis (18). From this database, athletes <50 years of age for whom CMR data were available were selected. Comparison was done with the male subjects of the current study.

### Cardiac magnetic resonance

CMR scans were performed on a 1.5T scanner. The CMR protocol consisted of localizers, a balanced steady-state free precession cine short-axis stack, and three long-axis views (4-, 2-, and 3-chamber views), and native T1 mapping and T2 mapping of 5–7 short-axis slices covering the left ventricle. Approximately 10 min after intravenous injection of 0.2 mmol/kg gadolinium, LGE imaging was performed of the left ventricular short-axis stack and three apical long-axis views. All CMRs were analyzed using CVI42 software (Circle Cardiovascular Imaging). Ventricular volumes and function were derived from the short-axis cine stack. The ULL were evaluated as recommended (16). LGE was scored as present when seen in two orthogonal planes. LGE at the right ventricular insertion point was not regarded as significant myocardial fibrosis.

### Statistical analysis

All analyses were performed using SPSS® 27.0.1.0. Continuous data were evaluated for normal distribution using the Shapiro–Wilk test. Normally distributed data were compared with an independent sample *t*-test, otherwise the Mann–Whitney *U* test was used. Frequencies were compared with the Chi-square test or Fisher exact test as appropriate. A two-tailed *p* < 0.05 was considered statistically significant. All data are reported as mean [standard error of mean (SEM)] unless stated otherwise.

## Results

**Figure 1:**
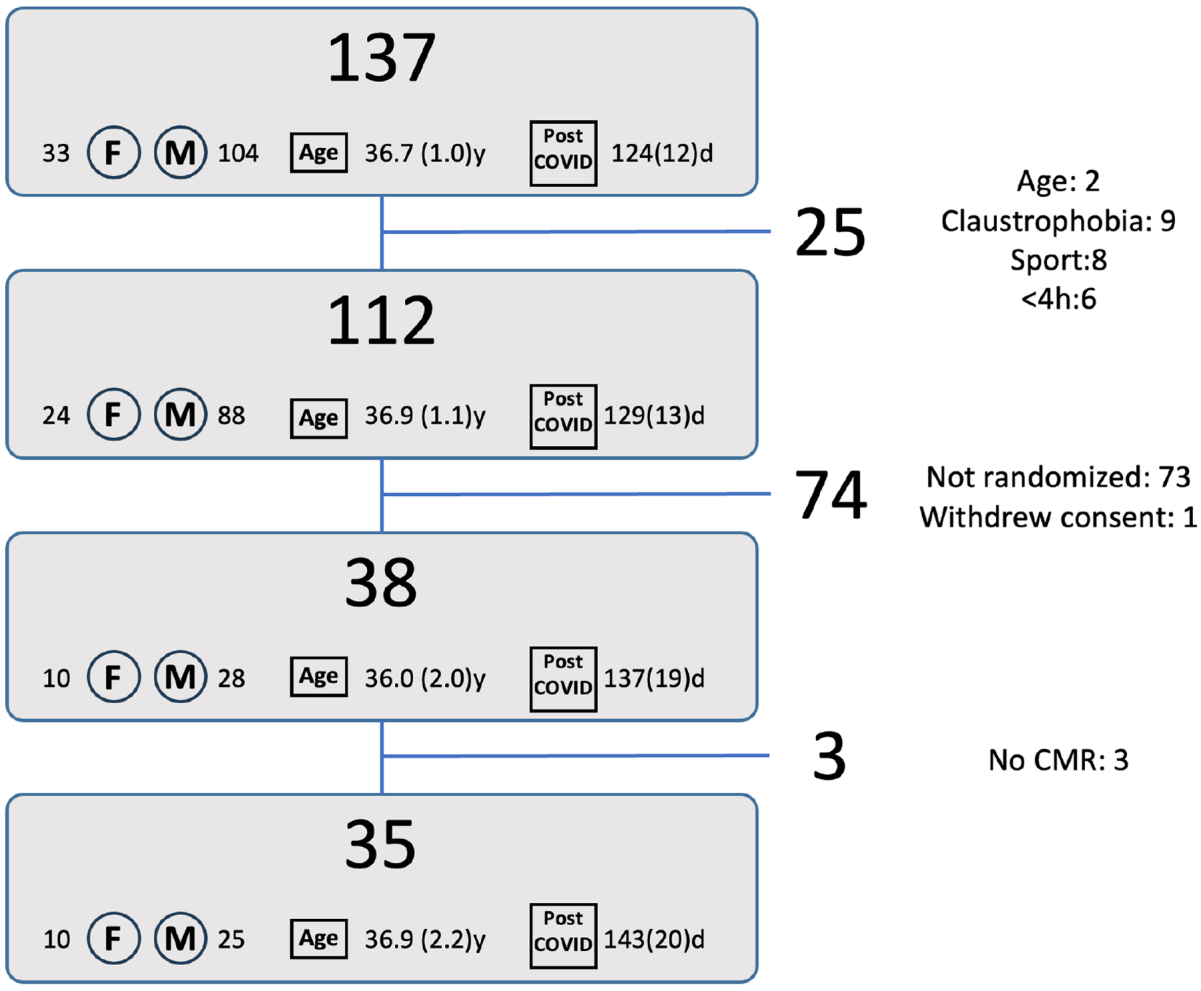
Flow diagram for patient inclusion. The numbers next to F and M represent the number of females/males, the number at “age” represent the average age [years(SE)], the numbers next to “post COVID” represent the time after initial diagnosis [days(SE)]. Reasons for exclusion are given in the right column: *Step 1*: age: 2 athletes were younger than 18 years, claustrophobia: 9 reported claustrophobia, sport: 8 did not perform an endurance or mixed type sport, <4 h: 6 athletes performed less than 4 h of sports per week. *Step 2:* Not randomized: 73 athletes were not randomized to undergo the study related tests. Withdrew consent: 1 athlete withdrew consent after randomization. *Step 3:* No CMR: 3 randomized athletes eventually did not undergo a CMR.

Between 9 March and 12 August 2021, a total of 137 individuals registered via the website. Registration occurred 124 ± 12 days (median 72, range 0–624 days) after SARS-CoV-2 infection, the mean age of the registrants was 36.7 ± 1.0 years, and 33 (24%) were women. After filtering for exclusion criteria (step 1, Figure 1) 112 patients were withheld for potential randomization. Of those, 39 were randomized following the protocol. One of those withdrew consent, hence 38 were scheduled for testing. Three of those eventually did not undergo CMR, resulting in inclusion of 35 patients in the final analysis. Of these, 18 athletes (4 female—22%) belonged to Group 1, 15 (6 female—40%) to Group 2, and 2 (both male) to Group 3.

Table 1 summarizes the symptoms at the time of diagnosis. In Group 1, 4 athletes (22%) were asymptomatic, while 14 (78%) showed mild symptoms (8 fatigue, 7 anosmia/dysgeusia, 6 cough, 5 myalgia, 3 headache, 3 fever, and 3 chills). In Groups 2 and 3, none were asymptomatic (per protocol). In Group 2, seven (47%) had cardiac symptoms (five chest pain, four dyspnea, one presyncope, and two palpitations).

**Table 1:** Symptoms at time of diagnosis.

Group	n	No symptoms	Anosmia dysgeusia	Fever	Cardiac symptoms	Chest Pain	Dyspnea	(Pre)syncope	Palpitations
1	18	4 (22%)	7 (39%)	3 (17%)	0 (0%)	0 (0%)	0 (0%)	0 (0%)	0 (0%)
2	15	0 (0%)	4 (27%)	8 (53%)	7 (47%)	5 (33%)	4 (27%)	1 (7%)	2 (13%)
3	2	0 (0%)	1 (50%)	0 (0%)	1 (50%)	1 (50%)	1 (50%)	0 (0%)	0 (0%)
	*p*	0.143	0.641	0.059	0.001	0.009	0.025	0.486	0.289

Numbers express the number of athletes (percentage of group) with the specific symptom at the time of diagnosis.

For further analysis Groups 2 and 3 were merged, combining moderate-to-severe symptomatic patients. Table 2 summarizes the baseline characteristics of these groups, demonstrating no statistically significant differences. Patients in Group 1 had a mean age of 35.2 ± 3.0 years, 14 (78%) were male and average body mass index was 22.5 ± 0.5 kg/m^2^. In Groups 2–3, the average age was 38.7 ± 3.2 years, 11 (65%) were men and the average body mass index was 22.6 ± 0.6 kg/m^2^ (all *p* = NS). Group 1 athletes performed an average of 9.6 ± 1.0 h of sports per week, while in Groups 2–3 this was 8.8 ± 1.1 h per week (*p* = 0.560). Group 1 contained a single former smoker (who quit smoking for more than 20 years), while in Groups 2–3, there were four former smokers (three had quit less than 1 year before inclusion, one more than 20 years). None of the athletes were current smokers. Overall, no differences in the presence of cardiac risk factors were found.

**Table 2:** Baseline characteristics.

	Group 1 (*n* = 18)	Groups 2–3 (*n* = 17)	*p*
Days post diagnosis (d)	158 (28)	126 (28)	0.429
Age (year)	35.2 (3.0)	38.7 (3.2)	0.421
Male [*n* (%)]	14 (78%)	11 (65%)	0.471
Height (m)	1.79 (0.01)	1.75 (0.02)	0.197
Weight (kg)	72.4 (2.3)	70.4 (2.6)	0.570
BMI (kg/m²)	22.5 (0.5)	22.6 (0.6)	0.856
BPsys (mmHg)	126.7 (2.9)	119.7 (3.2)	0.110
BPdia (mmHg)	76.3 (2.1)	73.8 (2.3)	0.423
Cardiac risk factors [*n* (%)]			
Smoking	1 (6%)	4 (24%)	0.177
Obesity	0 (0%)	0 (0%)	1
Hypertension	0 (0%)	1 (6%)	0.486
Family history	5 (28%)	6 (35%)	0.725
Diabetes	0 (0%)	0 (0%)	1
Lipids	4 (22%)	3 (18%)	1
Sport participation (h/w)			
Endurance	8.7 (1.1)	8.1 (1.1)	0.691
Mixed	0.9 (0.6)	0.7 (0.3)	0.798
Total	9.6 (1.0)	8.8 (0.9)	0.560

BPsys, systolic blood pressure; BPdia, diastolic blood pressure; data are expressed as mean (SE), unless stated otherwise.

### Primary outcome measures

#### Signs of myocarditis on CMR

In Group 1, CMR was performed 245 ± 27 days and in Groups 2–3, 209 ± 29 days after the diagnosis of SARS-CoV-2 infection (*p* = 0.384; overall range 35–499). The CMR-results are summarized in Table 3. The patients had generally mildly dilated left and right ventricles, as is commonly observed in athletes, without differences between Groups 1 and 2–3. None of the patients demonstrated signs of acute myocarditis based on the updated Lake Louise criteria. There were no patients with regional wall motion abnormalities of the left or right ventricle.

**Table 3:** CMR findings.

	Group 1 (*n* = 18)	Groups 2–3 (*n* = 17)	*p*
CMR days postdiagnosis (days)	245 (27)	209 (29)	0.384
LVEDVi (mL/m^2^)	113 (4)	105 (4)	0.173
LVSVi (mL/m^2^)	64 (3)	59 (2)	0.194
LVEF (%)	56 (1)	57 (1)	0.694
RVEDVi (mL/m^2^)	114 (4)	108 (5)	0.322
RVSVi (mL/m^2^)	62 (3)	57 (2)	0.166
RVEF (%)	54 (1)	53 (2)	0.692
Positive Lake Louise Criteria [*n* (%)]	0 (0%)	0 (0%)	1
T1-method (T1 mapping and/or LGE)	1 (6%)	2 (12%)	0.603
T2-method (T2 mapping and/or STIR)	0 (0%)	0 (0%)	1
Late gadolinium enhancement [*n* (%)]	1 (6%)	2 (12%)	0.603
Subendocardial	0	0	
Midwall	1	1	
Anteroseptum/anterior wall	0	0	
Inferoseptum/inferior wall	0	1	
Inferolateral/lateral wall	1	1	
Subepicardial	0	1	
Anteroseptum/anterior wall	0	0	
Inferoseptum/inferior wall	0	1	
Inferolateral/lateral wall	0	0	
Pericardial	0	0	
Pericardial effusion > physiological [*n* (%)]	0 (0%)	0 (0%)	1

LVEDVi, left ventricular end diastolic volume; i, indexed to body surface area (BSA); LVSV, left ventricular systolic volume; LVEF, left ventricular ejection fraction; RVEDV, right ventricular end diastolic volume; RVSV, right ventricular systolic volume; RVEF, right ventricular ejection fraction. Data are expressed as mean (SE), unless stated otherwise.

**Figure 2:**
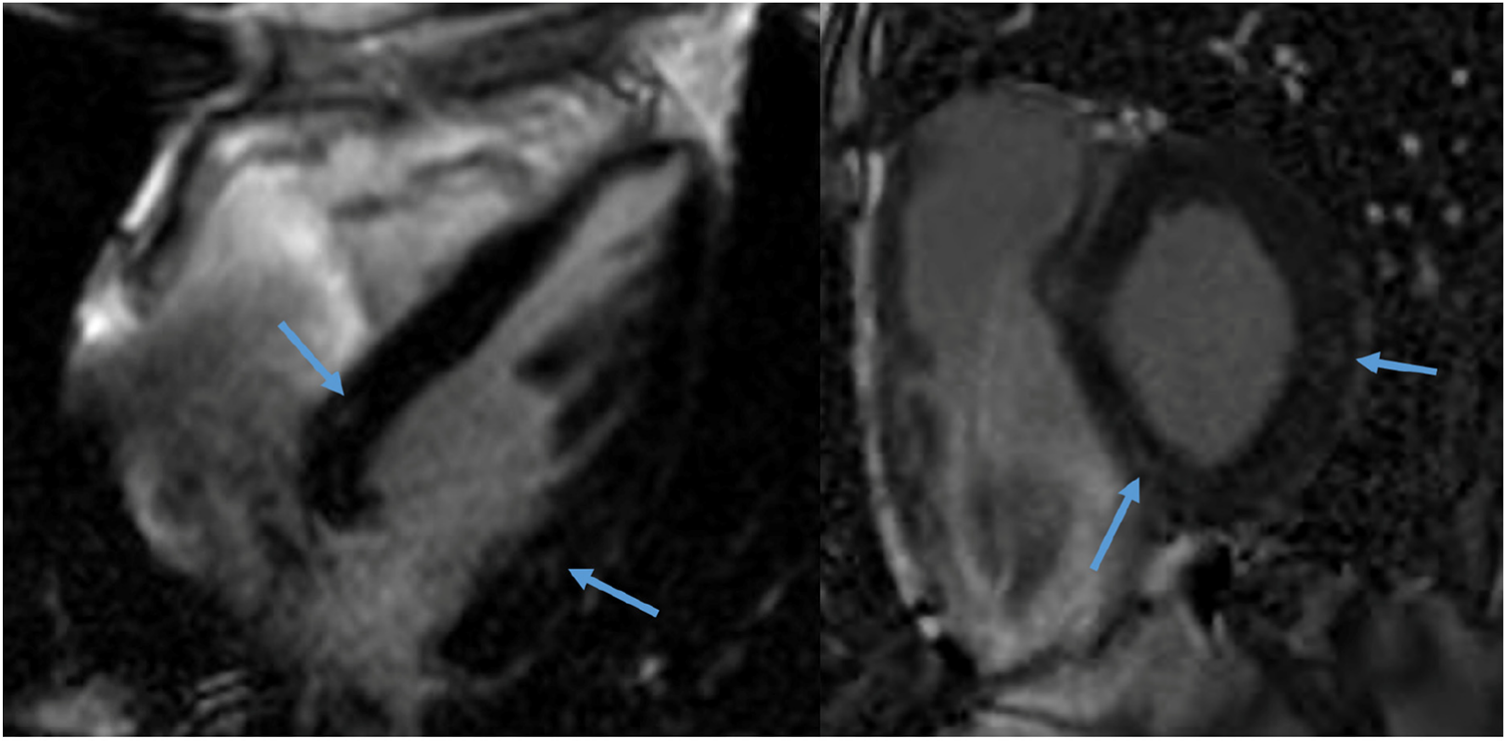
CMR of a 58-year-old male recreational athlete showing LGE. The late gadolinium enhancement (LGE) imaging showed two very small foci of midwall LGE (1.3% of total left ventricular mass) in the basal inferoseptum and basal (infero) lateral wall (arrows).

In Group 1, one male patient (6%) showed subtle midwall LGE of the basal inferolateral wall. In Groups 2–3, two male patients (12%) showed LGE: one patient had two small foci of midwall LGE in the basal inferoseptum and basal inferolateral wall (Figure 2), and one had subepicardial LGE of the basal inferolateral wall. There was no statistically significant difference in the presence of LGE between the two groups (*p* = 0.603). Inferior hinge point fibrosis was present in three athletes (8.5%): one athlete in Group 1 and two in Groups 2–3 (*p* = 0.603).

#### Arrhythmias

Both in Group 1 and Groups 2–3, 1 single athlete did not undergo Holter monitoring, resulting in 17 patients in Group 1 and 16 patients in Group 2–3 (Table 4). In Group 1, Holter monitoring was performed 280 ± 26 days after the diagnosis of SARS-CoV-2 infection, whereas the time interval was 214 ± 33 days in Groups 2–3 (*p* = 0.120). Group 1 patients had a median number [interquartile range (IQR)] of Ventricular ExtraSystoles (VES)/24 h of 1.6 (7). In Groups 2–3, this was 2.4 (15; *p* = 0.664). The range of VES/24 h was 0–492 in Group 1 and 0–513 in Groups 2–3. There was also no significant difference between the number of patients with more than 100 VES/24 h, more than 500 VES/24 h, patients with ventricular couplets, and patients with ventricular triplets. Only one Group 1 patient showed a non-sustained ventricular tachycardia (14 beats of monomorphic ventricular tachycardia during the night with a QRS duration of 120 ms and a very short RS_nadir_). This was a 41-year-old asymptomatic female cyclist with a total number of VES of 278 over 5 d and an entirely normal CMR. Similarly, no significant differences could be found in the occurrence of atrial extrasystoles between the two groups. In 1 Groups 2–3 patient, atrial fibrillation was detected. This patient had a history of atrial fibrillation already before the pandemic.

**Table 4:** Holter findings.

	Group 1 (*n* = 17)	Groups 2–3 (*n* = 16)	*p*
Holter monitoring days postdiagnosis (days)	280 (26)	214 (33)	0.120
HR average (BPM)	70 (2)	67 (2)	0.210
HR max (BPM)	171 (5)	160 (5)	0.155
HR min (BPM)	42 (2)	38 (6)	0.057
SVES/24 h^a^	0.6 (4.5)	1.0 (3.3)	0.914
AF > 30 s [*n* (%)]	0 (0)	1 (6)	0.485
VES/24 h^a^	1.6 (7)	2.4 (15)	0.664
>100 VES/24 h [*n* (%)]	1 (6)	3 (19)	0.335
>500 VES/24 h [*n* (%)]	0 (0)	1 (6)	0.485
Couplets [*n* (%)]	6 (36)	1 (6)	0.085
Triplets [*n* (%)]	2 (12)	1 (6)	1
NSVT [*n* (%)]	1 (6)	0 (0)	1

HR, heart rate; max, maximum; min, minimum; BPM, beats per minute; SVES, supraventricular extrasystoles; 24 h, 24 hours; AF, atrial fibrillation; VES, ventricular extrasystoles; >100 VES/24 h, number of athletes with more than 100 VES/24 h; >500 VES/24 h, analogous; couplets, triplets; NSVT, number of athletes (% of group) with at least one couplet, triplet, or non-sustained VT episode. Data are expressed as mean (SE), unless stated otherwise.

^a^ Median (IQR).

### Secondary outcome measures

#### Electrocardiogram

All patients were in sinus rhythm at presentation. Two patients showed an incomplete right bundle branch block (one in each group). There were no patients with pathological ST-segment alterations. Three Groups 2–3 patients showed T-wave inversion (TWI) beyond V2. All three had normal CMRs (Table 5).

**Table 5:** ECG findings.

	Group 1 (*n* = 18)	Groups 2–3 (*n* = 17)	*p*
HR (BPM)	63 (2)	68 (3)	0.245
Sinus rhythm [*n* (%)]	18 (100%)	17 (100%)	1
PR-interval (ms)	146 (4)	150 (6)	0.548
QRS-interval (ms)	94 (2)	92 (2)	0.575
iRBBB [*n* (%)]	1 (6%)	1 (6%)	1
cRBBB [*n* (%)]	0 (0%)	0 (0%)	1
Patho ST-elevation [*n* (%)]	0 (0%)	0 (0%)	1
ST-depression [*n* (%)]	0 (0%)	0 (0%)	1
QTc (ms)	403 (8)	417 (4)	0.125
TWI V3 [*n* (%)]	0 (0%)	3 (19%)	0.104
TWI V4 [*n* (%)]	0 (0%)	2 (13%)	0.229
VA [*n* (%)]	0 (0%)	0 (0%)	1

iRBBB, incomplete right bundle branch block; cRBBB, complete right bundle branch block; TWI, T-wave inversion; VA, ventricular arrhythmias. Other abbreviations as in the previous tables. Data are expressed as mean (SE), unless stated otherwise.

#### Cardiac troponin I levels

Mean hsTnI was 10.5 ± 2.0 ng/mL in 15 Group 1 patients and 8.7 ± 4.4 ng/mL in 9 Groups 2–3 patients (*p* = 0.540). None of the patients had an elevated hsTnI.

#### Comparison with healthy athletes

Of the 558 individuals in the Master@Heart database, CMR data had been analyzed for 214 subjects at the time of the present study. Of those, 34 were <50 years of age and were included before 1 January 2020 (i.e., before the start of the pandemic). Of those, 21 performed ≥4 h of endurance exercise per week. This group was compared with the male subjects (*n* = 25) from our study.

#### Baseline characteristics

Master@Heart subjects were on average 10 years older than the current study patients (47.9 ± 2.8 years vs. 37.1 ± 2.2 years; *p* < 0.001) and performed 3 h of sports per week more [median (IQR) 12.0 (4.0) vs. 9.0 (6.0); *p* = 0.019]. There were no other significant differences between the two groups (Table 6).

**Table 6:** Baseline characteristics Covidex vs. M@H.

	Covidex (*n* = 25)	M@H (*n* = 21)	*p*
Age (years)	37.1 (2.8)	47.9 (2.2)	<0.001
Height (m)	1.80 (0.01)	1.82 (0.01)	0.250
Weight (kg)	75.2 (1.7)	77.7 (1.9)	0.323
BMI (kg/m²)	23.2 (0.4)	23.4 (0.4)	0.799
BPsys (mmHg)	123.5 (2.7)	122.2 (2.5)	0.734
BPdia (mmHg)	74.2 (1.8)	73.7 (2.7)	0.486
Cardiac risk factors [*n* (%)]			
Smoking	3 (12%)	0 (0%)	0.239
Obesity	0 (0%)	0 (0%)	1
Hypertension	1 (4%)	1 (5%)	1
Family history	9 (36%)	4 (19%)	0.325
Diabetes	0 (0%)	0 (0%)	1
Lipids	5 (20%)	4 (19%)	1
Sports (h/w)	9.0 (6.0)	12.0 (4.0)	0.019

Abbreviations as in previous tables.

#### Signs of myocarditis on CMR

As in the current series, none of the Master@Heart athletes was positive for the ULL criteria. LGE was present in three patients in both groups: *p* = 1.000). In the Master@Heart population, one athlete had midwall LGE in the basal inferoseptum and inferior wall, one athlete had LGE in the midinferolateral and lateral wall, and one athlete had subepicardial LGE in the inferior wall (Table 7). Hinge point fibrosis was present in eight Master@Heart athletes (23.5%), as compared with the three in the current study (8.5%; *p* = 0.110).

**Table 7:** CMR findings Covidex vs. Master@Heart.

	Covidex (*n *= 25)	Master@Heart (*n* = 21)	*p*
LVEDVi (mL/m^2^)	110 (4)	121 (4)	0.057
LVSVi (mL/m^2^)	61 (3)	63 (2)	0.598
LVEF (%)	56 (1)	53 (1)	0.011
RVEDVi (mL/m^2^)	113 (4)	126 (3)	0.011
RVSVi (mL/m^2^)	61 (2)	64 (2)	0.229
RVEF (%)	53 (1)	51 (1)	0.166
Positive Lake Louise Criteria [*n* (%)]	0 (0%)	0 (0%)	1
T1-method (T1 mapping and/or LGE) [*n* (%)]	3 (12%)	3 (14%)	
T2-method (T2 mapping and/or STIR) [*n* (%)]	0 (0%)	0 (0%)	
Late gadolinium enhancement [*n* (%)]	3 (12%)	3 (14%)	1
Subendocardial	0	0	
Midwall	2	2	
Anteroseptum/anterior wall	0	0	
Inferoseptum/inferior wall	1	1	
Inferolateral/lateral wall	2	1	
Subepicardial	1	1	
Anteroseptum/anterior wall	0	0	
Inferoseptum/inferior wall	0	1	
Inferolateral/lateral wall	1	0	
Pericardial	0	0	
Pericardial effusion > physiological [*n* (%)]	0 (0%)	0 (0%)	1

Abbreviations as in the previous tables.

In line with the higher number of hours of sports, the indexed end-diastolic ventricular volumes were higher (reaching statistical significance for the right ventricle) and left ventricular ejection fraction was significantly lower (51 ± 1% vs. 53 ± 1%) than in the Covidex group (*p* = 0.011).

#### Arrhythmias

In the Master@Heart athletes, average heart rate (HR) (63 ± 1 bpm vs. 69 ± 1 bpm; *p* = 0.003) and maximal HR (122 ± 5 as compared with 169 ± 4 bpm; *p* < 0.001) were significantly lower. There were no other significant differences between the cohorts (Table 8).

**Table 8:** Holter findings Covidex vs. Master@Heart.

	Covidex (*n* = 25)	Master@Heart (*n* = 21)	*p*
HR average (BPM)	69 (1)	63 (1)	0.003
HR max (BPM)	169 (4)	122 (5)	<0.001
HR min (BPM)	39 (1)	41 (1)	0.523
SVES/24 h^a^	0.6 (3.6)	0.9 (1.3)	0.834
AF > 30 s [*n* (%)]	1 (4)	0 (0)	1
VES/24 h^a^	1.6 (6)	2.0 (34)	0.281
>100 VES/24 h [*n* (%)]	2 (8)	2 (10)	1
>500 VES/24 h [*n* (%)]	0 (0)	1 (5)	0.457
Couplets [*n* (%)]	6 (24)	6 (29)	1
Triplets [*n* (%)]	3 (12)	0 (0)	0.105
NSVT [*n* (%)]	0 (0)	0 (0)	1

Abbreviations as in the previous tables.

^a^ Median (IQR).

## Discussion

In our series of recreational athletes with prior confirmed SARS-CoV-2 infection, we found no evidence of ongoing myocarditis, and no more detection of fibrosis or ventricular arrhythmias than in a comparable athletic pre-COVID-19 cohort, pointing to much lower cardiac involvement of COVID-19 in athletes than initially suggested.

### Myocarditis

None of the athletes fulfilled the ULL criteria for myocarditis on average of 227 ± 20 days after SARS-CoV-2 infection. Other series with similar numbers of athletes also did not find any ULL positive individuals (19–21). Subsequent larger series equally demonstrated lower prevalences than reported in the initial reports (0.6%‒2.3%) (22–24). The timing of the CMR in our series was considerably later than in the other reports (which had mostly median intervals of 30 days) (25). Also, our subjects had a higher average age (36.9 ± 2.2 years as opposed to 20‒23 years in most other studies) and were recreational athletes (as opposed to professional or college athletes in the other series). These factors have previously been identified as potential sources of variance in the identification of myocarditis in different series (23, 24). However, despite the differences found in the various studies, the synthesis of these data does suggest a low actual prevalence of myocarditis post the SARS-CoV-2 infection, although the gold standard diagnostic (endomyocardial biopsy) is rarely reported or employed (26).

### Late gadolinium enhancement

A non-ischemic pattern of LGE was present in 3/35 (8.5%) of athletes in our series. In the Master@Heart control group, 3/21 athletes (14%) showed such a pattern, which did not significantly differ from the Covidex athletes (*p* = 1.000). This is in line with previous findings: prior to the COVID-19 pandemic, a non-ischemic pattern of LGE was reported in 4.7%–6.8% of master athletes (15, 27). More recent data (post-COVID-19) on LGE mostly found a lower prevalence (0.3%–2.3%) (22, 23), although some reports found prevalences as high as 17% (21) or even 46% (4).

Data from previous work suggest that a higher athletic exposure might be an important factor explaining the differences found between the different series: in three studies either cumulative training years (28, 29) or the number of completed marathons were associated with a higher prevalence of LGE (29, 30). This was corroborated by a recent meta-analysis (31). Yet, the evidence regarding this matter is not unequivocal: other studies found a correlation with age (although age and training load per definition go hand in hand) (32) or no correlation at all (33, 34). Future studies are underway to shed more light on this matter, along with the clinical relevance of such LGE patterns (17).

Hinge point fibrosis was present in 23.5% of Master@Heart athletes, as compared with the 8.5% in the current study (*p* = 0.110). In previous series of mostly master athletes, such fibrosis has been identified in 20%–30% of athletes and is generally regarded as a physiological adaptation to exercise (35).

We found no differences between the mildly and the more severely symptomatic groups. This is in part corroborated by the findings of the Big Ten registry, where symptoms alone only identified 5/37 (13.5%) cases of myocarditis, suggesting that most instances of myocarditis were subclinical (i.e., without symptoms suggestive of myocarditis) (23). Interestingly, in the Outcomes Registry for Cardiac Conditions in Athletes (ORCCA) registry, cardiopulmonary symptoms during COVID-19 were linked to cardiac involvement with an odds ratio of 3.1 (24). Our series is too small to draw definitive conclusions.

Further comparison between the Covidex and Master@heart athletes revealed no additional relevant differences. Previous work already found that higher levels of endurance are associated with more ventricular dilation and lower ejection fractions at rest, findings that were confirmed in our analysis (36).

### Arrhythmias

Overall, arrhythmias were rare. One female athlete showed a non-sustained ventricular tachycardia of 14 beats, another female athlete showed more than 500 VES/24 h, and one male athlete with a history of atrial fibrillation had arrhythmia recurrences during the monitoring period. When compared with the Master@Heart data, no significant differences were found, except for a lower resting and maximum heart rate in the latter group. This can be expected from the 10-year age difference and higher exercise load. We were not able to analyze whether palpitations were associated with specific arrhythmias, because only two athletes experienced palpitations. One of them reported palpitations at rest and had 5 isolated VES, 1 ventricular triplet, and 403 single atrial extrasystoles over 5 days. The other reported palpitations during exercise and had 11 isolated VES and 525 single atrial extrasystoles over 5 days.

Although early reports suggested very high incidences of arrhythmic events (in hospitalized COVID-19 patients) (37), later reports have put these data into perspective, suggesting that the COVID-19 associated critical illness, rather than the SARS-CoV-2 infection itself, was the more likely cause of the observed arrhythmias (38). A study in an ambulatory post-COVID-19 population (median 75 days) did not observe a higher-than-expected arrhythmic burden (39). Interestingly, most incidences of palpitations in that study (78% of patient-recorded events) were associated with sinus rhythm or sinus tachycardia. The authors stated in a different publication that “there is presently no definitive data to establish a causal, viral-specific association between COVID-19 and incident arrhythmia” (40). Our results certainly fit with this conclusion.

### Secondary endpoints

Resting electrocardiograms were generally normal. TWI beyond V2 was found in three female athletes. Anterior TWI is more common in women and in athletes and does not necessarily hold prognostic significance (41). However, TWI beyond V3 is rare. All three athletes had normal CMRs and a negative family history.

No elevation in hsTnI was found in this population. This could be expected from the late postdiagnosis evaluation in our series.

Overall, we found no significant differences between Group 1 and Groups 2–3 athletes. This suggests that symptoms *per se* are not indicative of cardiac clinical consequences of an SARS-CoV-2 infection. This is in part corroborated by the findings of the Big Ten registry, where symptoms alone only identified 5/37 (13.5%) cases of myocarditis, suggesting that most instances of myocarditis were subclinical (i.e., without symptoms suggestive of myocarditis) (23).

### Limitations

After publication of the ORCCA registry with confirmation of the lower than previously feared prevalence of myocarditis post-COVID-19 (24), we decided to stop testing more individuals, as the original power calculation was based on a 20% prevalence. This resulted in an underpowering of our study. Nevertheless, we found no presence of ULL criteria, thus confirming the more recent findings, and found it worthwhile to still report our findings to add to the literature.

The study was observational in nature, so no conclusions can be drawn about a causal effect of SARS-CoV-2 on our findings (of e.g., non-ischemic LGE). The similarities between the Covidex and Master@Heart (a non-SARS-CoV-2 exposed population), however, seem to indicate that SARS-CoV-2 by itself does not pose a particular threat for myocarditis in athletes.

We may have underdiagnosed SARS-CoV-2-related myocarditis in our series because of the later timing of CMR. This was a deliberate choice as we aimed to gain an insight into the potential late sequelae of COVID-19: fibrosis may predispose to arrhythmias, which are of particular concern in athletes. Fortunately, myocarditis is known to heal without sequelae in most patients. In the Big Ten registry, 40.7% of athletes with signs of COVID-19 myocarditis showed complete resolution of CMR abnormalities 8 (4–10) weeks after the first CMR, which had been taken within a month from the initial diagnosis. The remaining athletes showed resolution of the edema (T2 criteria) but not LGE after 12 (4–14) weeks (23). In the ORCCA registry, 70% showed complete resolution, 10% resolution of T2 abnormalities, and 20% showed persistent positive ULL criteria on repeat CMR (42).

### Public health implications

Although COVID-19 can cause serious illness and carry major complications, in general the course of the disease is benign, with 0.05% adverse cardiac events in a recent series of young competitive athletes (43). This implies that general rules regarding return-to-play after viral disease can also be applied to COVID-19 (44): the infection must be cleared, without any remaining symptoms and the process should be gradual and closely monitored. Athletes need to be reminded that aerobic capacity may be decreased for prolonged periods of time after SARS-CoV-2 infection (45). Further work-up is specifically warranted when exercise-related cardiac symptoms, especially chest pain, persist beyond 3-week postinfection (7). Within these contours, an individualized approach may be adapted when reasons are clear, after informed decision-making, preferably with all parties involved reaching a consensus. Our data extend the evidence of a low cardiac involvement to recreational athletes that were older than those in the previous series. Thus, although ongoing vigilance is warranted, a similar return-to-play approach is warranted in this population.

## Conclusion

In our series of recreational athletes, we found no evidence of ongoing myocarditis or increases in myocardial fibrosis on average 6 months after SARS-CoV-2 infection. Also, the arrhythmic burden was comparable with historical controls. The results from CMR and heart rhythm monitoring were similar between the asymptomatic or mildly symptomatic athletes and the moderately to severely symptomatic groups.

The results of our series add to other reports with reassuring findings in athletes post-COVID-19. Together, these observations can provide guidance in counseling athletes with an SARS-CoV-2 infection history.

## Data Availability

The raw data supporting the conclusions of this article will be made available by the authors, without undue reservation.
